# Protocol for the perioperative outcome risk assessment with computer learning enhancement (Periop ORACLE) randomized study

**DOI:** 10.12688/f1000research.122286.2

**Published:** 2022-09-29

**Authors:** Bradley Fritz, Christopher King, Yixin Chen, Alex Kronzer, Joanna Abraham, Arbi Ben Abdallah, Thomas Kannampallil, Thaddeus Budelier, Arianna Montes de Oca, Sherry McKinnon, Bethany Tellor Pennington, Troy Wildes, Michael Avidan

**Affiliations:** 1 Department of Anesthesiology, Washington University School of Medicine, St. Louis, Missouri, 63110, USA; 2 Department of Computer Science and Engineering, Washington University McKelvey School of Engineering, St. Louis, Missouri, 63130, USA; 3 Institute for Informatics, Washington University School of Medicine, St. Louis, Missouri, 63110, USA

**Keywords:** Anesthesiology, Machine Learning, Postoperative Complications, Protocol, Surgery

## Abstract

**Background:** More than four million people die each year in the month following surgery, and many more experience complications such as acute kidney injury. Some of these outcomes may be prevented through early identification of at-risk patients and through intraoperative risk mitigation. Telemedicine has revolutionized the way at-risk patients are identified in critical care, but intraoperative telemedicine services are not widely used in anesthesiology. Clinicians in telemedicine settings may assist with risk stratification and brainstorm risk mitigation strategies while clinicians in the operating room are busy performing other patient care tasks. Machine learning tools may help clinicians in telemedicine settings leverage the abundant electronic health data available in the perioperative period. The primary hypothesis for this study is that anesthesiology clinicians can predict postoperative complications more accurately with machine learning assistance than without machine learning assistance.

**Methods:** This investigation is a sub-study nested within the TECTONICS randomized clinical trial (NCT03923699). As part of TECTONICS, study team members who are anesthesiology clinicians working in a telemedicine setting are currently reviewing ongoing surgical cases and documenting how likely they feel the patient is to experience 30-day in-hospital death or acute kidney injury. For patients who are included in this sub-study, these case reviews will be randomized to be performed with access to a display showing machine learning predictions for the postoperative complications or without access to the display. The accuracy of the predictions will be compared across these two groups.

**Conclusion:** Successful completion of this study will help define the role of machine learning not only for intraoperative telemedicine, but for other risk assessment tasks before, during, and after surgery.

**Registration: **ORACLE is registered on ClinicalTrials.gov: NCT05042804; registered September 13, 2021.

## Introduction

### Background and rationale

Each year, more than four million people worldwide die within 30 days after surgery, making perioperative events the third-leading cause of death. ^1^ One common postoperative complication that is associated with an increased risk of death is acute kidney injury (AKI). ^2^^–^^6^ In one retrospective study, postoperative AKI occurred in 39% of hospitalized surgical patients who had their creatinine checked after surgery. ^7^ In-hospital mortality increased from 0.6% among those without AKI to 8.8% among those who experienced AKI.

Some postoperative deaths and AKI may be prevented through identification and modification of risk factors. Although some risk factors (e.g., age ^8^^–^^10^) are not modifiable and other risk factors (e.g., preoperative glycemic control ^8^^,^^11^^,^^12^) are no longer modifiable once surgery has begun, several important risk factors are under the anesthesiologist’s control during surgery. For example, intraoperative hypotension is a well-established risk factor both for postoperative death ^13^^–^^15^ and for postoperative AKI. ^15^^–^^17^ Interventions on such risk factors may therefore affect outcomes. ^18^^,^^19^ Appropriate adjustments to the postoperative care plan may also impact outcomes. ^19^

Telemedicine has successfully improved mortality and other outcomes in the intensive care unit (ICU), and similar results may be expected in the operating room. In a stepped-wedge trial across units in a single medical center, tele-ICU implementation was associated with an absolute mortality reduction of nearly 2%. ^20^ In a subsequent multi-center pre-post study, tele-ICU was associated with reduced in-hospital mortality and length of stay. ^21^ Improved outcomes were associated with enhanced compliance with best clinical practice guidelines. ^20^^,^^21^ The effect of telemedicine on outcomes may depend on characteristics of the patients in question—in one study, tele-ICU implementation improved mortality only among patients with higher illness severity scores. ^22^

The effectiveness of a telemedicine intervention depends on how well clinicians working in the telemedicine setting can assess patient risk and identify potential interventions to reduce risk. Accurate intraoperative risk assessment by clinicians is challenging for the following reasons. First, the sheer volume of data available is more than the human brain can comprehend with its limited data processing capacity. ^23^^,^^24^ Competing clinical demands reduce the cognitive capacity available to process these data. ^25^^,^^26^ Second, anesthesiology clinicians frequently use the available data to make decisions that are not based on sound logic. ^27^ Clinicians may anchor on the first diagnosis that comes to mind, only seek out data that confirm a previously suspected diagnosis, or interpret ambiguous findings with a false sense of certainty. ^27^ These concerns become increasingly significant as clinicians monitor increasing numbers of patients and have less time to review each patient’s information.

Clinicians in telemedicine settings may use machine learning (ML) tools to address their innate cognitive deficiencies. Computers can process larger quantities of data more quickly than a human, model more complex relationships and interactions among inputs, and will not fatigue. ^28^^–^^30^ The ability of clinicians to integrate the output of ML tools into their overall assessment of the patient depends on the ML output being presented in a manner that makes sense to the clinician and caters to the clinician’s information needs. ^31^^,^^32^ These needs will vary depending on the clinician’s background and the context in which the clinician is working.

### Preliminary data

This study is possible because the investigators have previously developed ML algorithms for predicting postoperative death and AKI. They used a retrospective cohort of approximately 110,000 patients who underwent surgical procedures at Barnes-Jewish Hospital between 2012 and 2016. ^33^ This dataset was divided into a training set (for model parameter tuning), a validation set (for hyperparameter tuning), and a test set (for quantifying model performance) in a ratio of approximately 7:1:2. Inputs to the models included patient characteristics, health conditions, preoperative vital signs and laboratory values, and intraoperative vital signs and medications. The resulting model predicted postoperative death with excellent accuracy (area under the receiver operating characteristic curve of 0.91, 95% confidence interval 0.90-0.92). ^34^^,^^35^ A separate model predicted AKI with good accuracy (area under the receiver operating characteristic curve of 0.82, 95% confidence interval 0.81-0.84). ^36^

In addition, the investigators have recently conducted a series of focus groups and user interviews with anesthesiology clinicians (unpublished data) to learn about their workflows and information needs when working in the intraoperative telemedicine unit at Barnes-Jewish Hospital. Based on these insights, the investigators have designed a display interface that shows ML predictions for postoperative death and AKI in real-time during surgery.

### Objective

To determine whether anesthesiology clinicians in a telemedicine setting can predict postoperative death and AKI more accurately with ML assistance than without ML assistance. The hypothesis is that clinician predictions will be more accurate with ML assistance than without ML assistance.

### Overall study design

The Perioperative Outcome Risk Assessment with Computer Learning Enhancement (Periop ORACLE) study will be a sub-study nested within the ongoing TECTONICS trial (NCT03923699). TECTONICS is a single-center randomized clinical trial assessing the impact of an anesthesiology control tower (ACT) on postoperative 30-day mortality, delirium, respiratory failure, and acute kidney injury. ^37^ As part of the TECTONICS trial, clinicians in the ACT perform medical record case reviews during the early part of surgery and document how likely they feel each patient is to experience postoperative death and AKI. In Periop ORACLE, these case reviews will be randomized to be performed with or without access to ML predictions.

## Methods: Participants, interventions, and outcomes

### Study setting

The study will be conducted at Barnes-Jewish Hospital, a 1,252-bed university-affiliated tertiary care facility in St. Louis, MO. About 19,000 inpatient surgeries are performed in the hospital’s 58 operating rooms each year.

### Eligibility criteria

The participants will include all patients enrolled in the TECTONICS trial during the 12-month sub-study period for whom the ACT clinicians conduct a case review. The inclusion criteria for TECTONICS include (1) surgery in the main operating suite at Barnes-Jewish Hospital, (2) surgery during hours of ACT operation (weekdays 7:00am-4:00pm), and (3) age ≥ 18. Exclusion criteria include procedures performed without anesthesiology services.

### Recruitment

Participants are currently enrolled in TECTONICS via a waiver of consent. The investigators have obtained a waiver of informed consent to include these patients in Periop ORACLE as well.

### Interventions – Machine learning algorithms

The machine learning models used in this study were originally trained and validated on a retrospective cohort of approximately 110,000 adult patients who underwent surgery with general anesthesia at Barnes-Jewish Hospital between 2012 and 2016. Input features included demographic characteristics, comorbid conditions, preoperative vital signs, surgical service, functional capacity as documented during the preoperative assessment, and most recent values of selected laboratory tests. A random forest model was implemented in scikit-learn. In the holdout validation cohort of 21,171 patients, the incidence of postoperative death was 2.2% and the model predicted this outcome with receiver operating characteristic area under curve (AUC) of 0.939 and precision-recall AUC of 0.161. The incidence of postoperative AKI was 6.1% and the model predicted this outcome with receiver operating characteristic AUC of 0.799 and precision-recall AUC of 0.275.

In February 2022, the models were retrained using a newer cohort of 84,455 patients who underwent surgery with general anesthesia at Barnes-Jewish Hospital between 2018 and 2020. This time period was after the hospital had transitioned from its previous electronic health record systems (including MetaVision as its anesthesia information management system) to Epic (Epic, Verona, WI). Input features were the same as the previous models, with the addition of the planned surgical procedure text field. A regularized logistic regression was used to predict the outcome from the words in the planned surgical procedure text field. This was used to initialize a gradient boosted decision tree, which was trained using the remaining features. Hyperparameters were selected by 10-fold cross validation. Models were implemented in XGBoost. In the holdout validation cohort of 16,891 patients, the incidence of postoperative death was 1.9% and the model predicted this outcome with receiver operating characteristic AUC of 0.91 and precision-recall AUC of 0.27. The incidence of postoperative AKI was 13.3% and the model predicted this outcome with receiver operating characteristic AUC of 0.90 and precision-recall AUC of 0.66.

A password-protected web application on a secure server has been created for delivery of machine learning algorithm outputs to clinicians. The design of this web application was informed by input obtained by users (clinicians from the ACT) in focus group sessions. For each patient, the machine learning predictions are presented in the form of predicted probabilities of each outcome (e.g., 4.5% chance of AKI). In addition, a list of features contributing most to the prediction is shown, along with Shapley values. During the focus group meetings, most users said they would find it overwhelming to see confidence intervals around the predicted probabilities. A fact sheet about each model is available on demand, including the receiver operating characteristic curve, precision-recall curve, and calibration curve.

### Interventions – Implementation and workflow

Clinicians in the ACT currently conduct case reviews by viewing the patient’s records in AlertWatch (AlertWatch, Ann Arbor, MI) and Epic. AlertWatch is an FDA-approved patient monitoring system designed for use in the operating room. Clinicians in the ACT use a customized version of AlertWatch that has been adapted for use in a telemedicine setting. ^38^ Epic is the electronic health record system utilized at Barnes-Jewish Hospital. For patients included in Periop ORACLE, each case review will be randomized in a 1:1 fashion to be completed with or without ML assistance. If the case review is randomized to ML assistance, the clinician will access the machine learning web application (see previous section) during the case review. Study staff will be immediately available in the ACT during all case reviews to assist with accessing the display interface if needed to improve adherence to the protocol. If the case review is not randomized to ML assistance, the clinician will not access this web application. This choice of comparator mimics current practice more closely than using an active comparator. After viewing the patient’s data, the clinician will complete a case review from in AlertWatch (as described in the data collection section later in this document).

### Outcomes

The co-primary outcomes will be clinician accuracy in predicting postoperative death and clinician accuracy in predicting postoperative AKI. Clinician predictions will be retrieved from the case review forms completed in AlertWatch. Observed death and observed AKI will be retrieved from Epic. Death will be defined as 30-day in-hospital mortality. AKI will be defined as a creatinine increase ≥0.3 mg/dl above baseline within 48 hours or an increase to ≥1.5 times baseline within seven days, consistent with the kidney disease: improving global outcomes definition. ^39^ If no preoperative creatinine is available, then the upper limit of the laboratory’s reference range (1.2 mg/dl) will be used as the baseline.

Secondary outcomes will include AKI stage 2 or greater (creatinine increase to ≥2 times baseline within seven days) and AKI stage 3 (creatinine increase to ≥3 times baseline within seven days, an increase to ≥4.0 mg/dl, or initiation of renal replacement therapy).

### Participant timeline

No direct interactions between study staff and the participants are planned, and no anesthetic interventions will be prohibited based on participation in this trial. The initial medical record review and clinician predictions will occur during the participants’ surgery. Additional data retrieval to obtain observed death and AKI will occur at least 30 days after surgery (and may occur in bulk 30 days after the final participant’s surgery).

### Sample size

The sample size calculation is based on the assumption that ML-assisted clinicians would predict each outcome with receiver operating curve area under curve (AUC) similar to the published AUC of the ML algorithms. ^34^^,^^36^ Incidences of death and AKI were also taken from these previous publications. A simulation population of 100,000 patients was generated. ML-assisted and -unassisted clinician predictions were simulated with beta distributions whose parameters were adjusted to achieve the specified AUC. For each sample size tested, 1,000 random samples were drawn and the difference in AUC between the assisted and unassisted clinician predictions was determined. ^40^ Power at each sample size was defined as the fraction of samples for which the two sets of predictions had significantly different AUC at α = 0.025. The minimum clinically meaningful difference (MCMD) in AUC was defined as 0.07.

**Figure 1.**
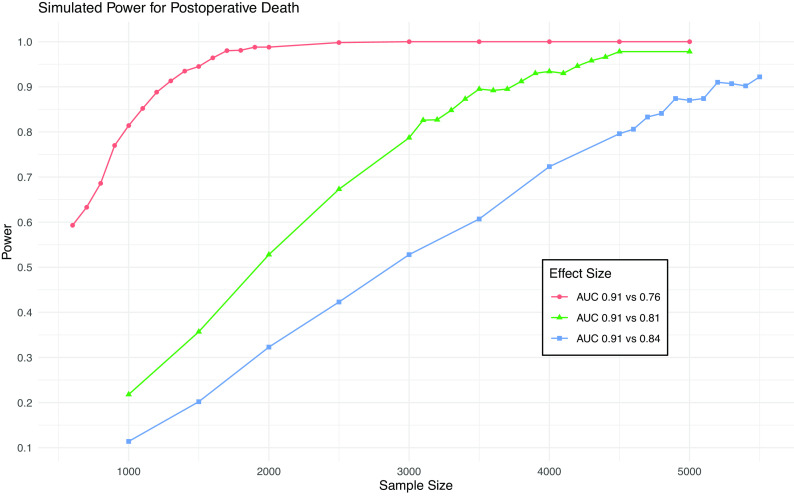
Simulation results from power calculation for death. Power achieved in simulations of various sample sizes and effect sizes for postoperative death. Blue line is minimum clinically meaningful difference.

**Figure 2.**
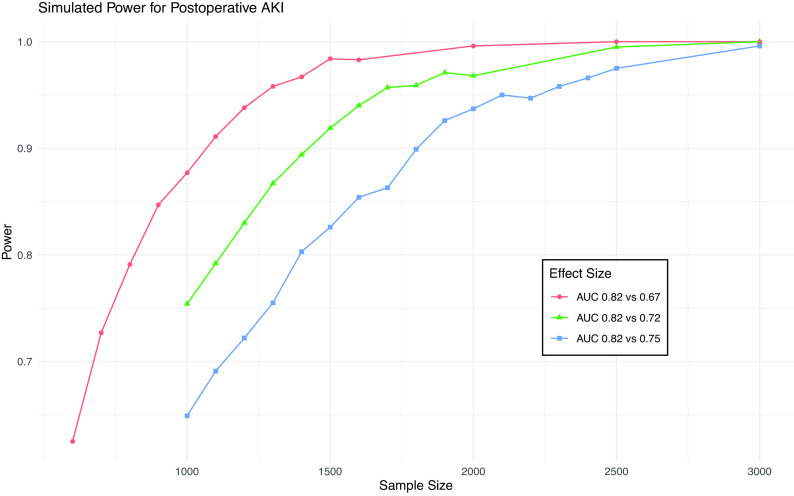
Simulation results from power calculation for AKI. Power achieved in simulations of various sample sizes and effect sizes for postoperative AKI. Blue line is minimum clinically meaningful difference.

As shown in Figure 1, a sample size of 4,500 will provide 80% power to detect a difference in AUC from 0.91 to 0.84 (the MCMD) for death and 95% power to detect a difference from 0.91 to 0.81. This sample size will give >99% power to detect a difference in AUC from 0.82 to 0.75 (the MCMD) for AKI ( Figure 2).

To allow for 15% missing data, we will enroll 5,300 cases. Currently, about 20 case reviews are performed in the ACT on a typical day. We should therefore be able to complete enrollment over a period of 12 months.

## Methods: Assignment of interventions

### Allocation

**Figure 3.**
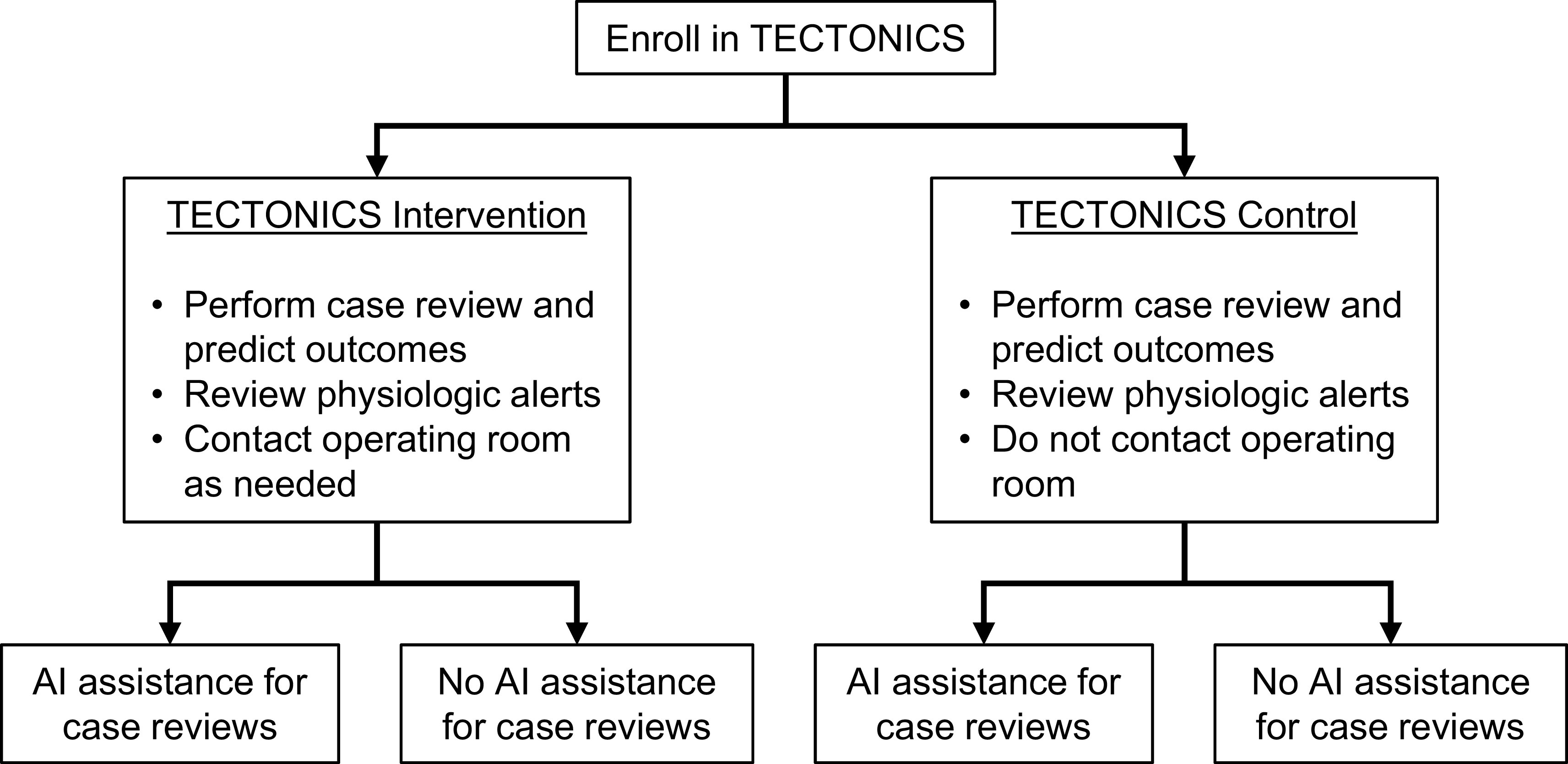
Flow chart showing treatment allocation. Periop ORACLE participants will be randomizated to ML assistance or no ML assistance, stratified by intervention or control status of the parent TECTONICS trial.

Each participant case review will be randomized in a 1:1 fashion to be completed with or without ML assistance. Randomization will be stratified by intervention/control status of the parent trial because clinicians may be biased to perform case reviews more carefully in the TECTONICS intervention group than in the TECTONICS control group ( Figure 3). The allocation sequence will be generated by computer-generated random numbers within the AlertWatch software. The allocation will be automatically displayed on the case review form when the clinician opens it. The study staff will not have access to the allocation sequence before the case review.

### Blinding

By necessity, the clinician completing the case review and the other study staff in the ACT will not be blinded to treatment allocation. Study personnel who retrieve observed postoperative complications from the electronic health record will be blinded.

## Methods: Data collection, management, and analysis

### Data collection

**Figure 4.**
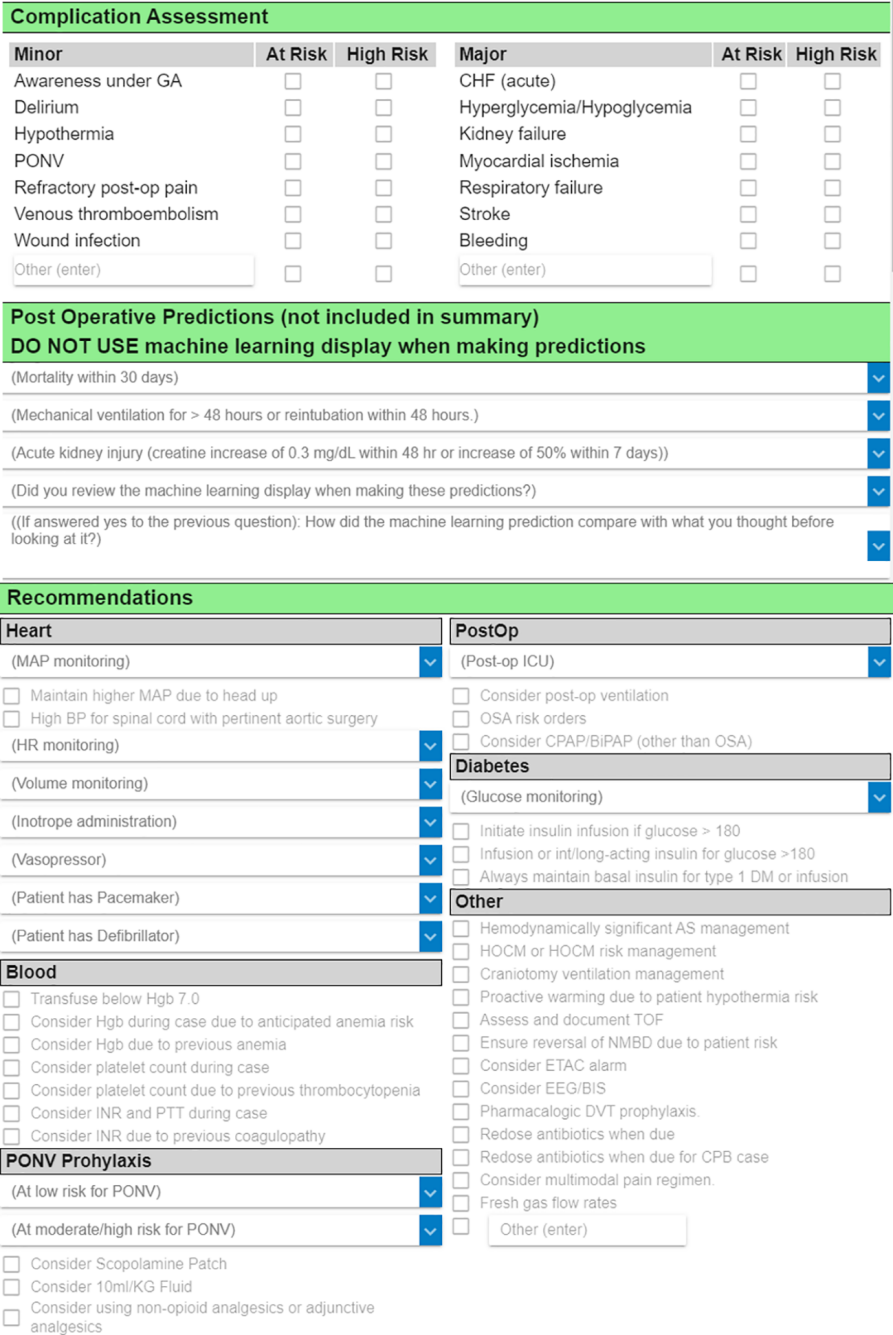
Case review form in AlertWatch. The first two sections contain fields for documentation of clinician predictions of postoperative complications, and the Periop ORACLE randomization allocation is disclosed in the header of the second section. The treatment recommendations documented in the third section are utilized for the parent TECTONICS trial but not for this sub-study. The amount of time the clinician in the ACT spends completing each case review will be retrieved from the Epic audit log.

Clinician predictions will be collected via an existing case review form in AlertWatch that contains two sections ( Figure 4). In the first section, the clinician documents how likely the patient is to experience postoperative death within 30 days, postoperative AKI within seven days, and a few other complications. The clinician selects their choice from a five-point ordered categorical scale (very low risk, low risk, average risk, high risk, and very high risk). In the second section (which is part of the parent TECTONICS trial but not used for this sub-study), the clinician selects treatment recommendations (e.g., blood pressure goals, medications to administer or avoid). An additional question asks the clinician to confirm whether they reviewed the ML display interface prior to documenting their predictions in section one. Finally, clinicians are asked whether the ML predictions on the display interface agree with their previous opinion and whether the display interface caused the clinician to change their opinion.

Observed death and observed AKI will be retrieved from Epic via a query of the Clarity database. Observed outcomes will be retrieved for all randomized participants, including those with protocol deviations.

### Data management

The clinical applications used in this project (AlertWatch and Epic) can only be accessed over the secure institutional internet network or using virtual private network, and user authentication is required for access. Computations for the ML models will occur on institutional servers that meet the standards of the Health Insurance Portability and Accountability Act. The ML display interface is also hosted on an institutional server, can only be accessed over the secure institutional internet network or using virtual private network, and requires user authentication for access. For data analysis, the amount of protected health information retrieved will be limited to the minimum amount necessary to achieve the aims of the study. All study data will be stored on institutional servers, and access will be limited to study personnel.

### Statistical methods

To compare the accuracy of clinician predictions with and without ML assistance, the investigators will construct logistic regressions for death and for AKI. Separate models will be constructed for the ML-assisted and -unassisted groups. The only inputs will be dummy variables encoding the clinician predictions ( Table 1). The regression coefficients will be restricted to positive values, thus modeling a monotonic increasing relationship between the clinician predictions and the true incidence of death and AKI. The AUC of the model constructed from ML-assisted cases will be compared to the AUC of the model constructed from ML-unassisted cases, ^40^ using the Holm method to ensure the family-wise error rate remains less than a two-sided α = 0.05 across the two co-primary outcomes—accuracy of death prediction and accuracy of AKI prediction. The null hypothesis is that the AUCs will be equal.

**Table 1.** Dummy variable encoding for clinician predicted risk of postoperative death.

Clinician prediction	Var1	Var2	Var3	Var4
Very low	0	0	0	0
Low	1	0	0	0
Average	1	1	0	0
High	1	1	1	0
Very high	1	1	1	1

The primary analysis will follow an intention-to-treat principle, and all case reviews will be included in the group to which they were randomized. Patients on dialysis preoperatively, undergoing dialysis access procedures, or with baseline creatinine > 4.0 mg/dl will be excluded from the AKI analysis but included in the death analysis. In a secondary per-protocol analysis, case reviews will be grouped according to whether the clinician reported viewing the ML display or not. Exploratory analyses stratified by sex and race will evaluate for biases learned during training. ^41^ If either clinician predictions or observed outcomes are missing for a given case, then the case will be excluded from the analysis. No interim analyses are planned. To determine whether different levels of clinician engagement in the TECTONICS intervention group versus the TECTONICS control group impact the findings, sensitivity analyses will be conducted stratified by TECTONICS intervention status. To examine the effects of dataset shift and model retraining, the prospective performance of the models will be reported for each month of the study, and sensitivity analyses will be conducted in the subgroups who had surgery before and after the February 2022 retraining event.

To examine human-computer agreement, the proportion of cases for which the clinician reported being surprised by the ML prediction will be determined. Among those cases for which the clinician was surprised, the proportion for which the clinician self-reported agreeing or disagreeing with the ML prediction will be determined.

To examine the potential impact of inaccurate ML predictions, we will conduct the following sensitivity analysis. First, the predicted probabilities output by the ML algorithms will be converted to dichotomous predictions by using the cutoff value that maximizes the Youden index. Second, the cases will be categorized as having correct or incorrect ML dichotomized predictions. Finally, the primary analysis will be repeated in the subgroup with correct ML predictions and in the subgroup with incorrect ML predictions.

To estimate the effect of the ML predictions on case review efficiency, chart review duration (approximated using the time the Epic chart was open, retrieved from the audit log) will be compared between case reviews performed with ML assistance and those performed without ML assistance, using either an unpaired T test or Wilcoxon rank sum test as appropriate.

## Ethics and dissemination

### Ethical statement

This study has been approved by the Human Research Protection Office at Washington University in St. Louis (approval #202108022) on August 26, 2021. Any protocol amendments will be approved by the study steering committee and communicated with the institutional review board. This study presents patients with minimal risks, other than a small risk for a breach of confidentiality if protected health information were to become unintentionally available to individuals outside the study team. To protect against this risk, all electronic data will be kept in an encrypted, password-protected environment accessible only to the research team (see Data Management section). Because the risks are minimal, no dedicated safety monitoring committee is planned for Periop ORACLE. However, the parent TECTONICS trial does have a safety monitoring committee. The institutional review board has granted a waiver of informed consent to enroll patients in this study.

Study results will be presented at national or international scientific meetings and published in a peer-reviewed publication. Individuals who meet the International Committee of Medical Journal Editors authorship guidelines ( https://www.icmje.org/recommendations/browse/roles-and-responsibilities/defining-the-role-of-authors-and-contributors.html) will be included as authors on any publications resulting from this work. To comply with data sharing recommendations, de-identified individual participant data underlying the study results will be made available to researchers who provide a methodologically sound proposal for utilizing that data.

### Strengths, limitations, and alternative strategies

This project has multiple strengths. First, this protocol has been prepared in accordance with Standard Protocol Items: Recommendations for Interventional Trials (SPIRIT) guidelines. ^42^^,^^43^ Second, the sample size of predictions made by experienced anesthesia clinicians will be large. The pragmatic design of superimposing Periop ORACLE on the established TECTONICS trial where many case reviews are already being conducted on a daily basis makes it feasible to achieve such a large sample size. Third, the ML models to be used have very good AUC, which enhances the likelihood that ML assistance can increase the accuracy of clinician predictions. Fourth, the ML display interface has been created using a human-centered design framework informed by the clinicians who work in the ACT, which maximizes the chances that clinicians will integrate the ML display interface into their workflows and their decision-making.

This project also has limitations. First, data collection in a telemedicine setting rather than in the operating room may limit the generalizability of the findings to institutions that do not utilize intraoperative telemedicine. However, multiple clinicians have stated during focus group interviews that their workflows for performing case reviews in the ACT closely mimic their workflows for preparing to provide bedside anesthesiology care. Thus, the findings may be relevant in the operating room as well. Second, this is a single-center study, so differences in patient population or practice patterns at other institutions may limit generalizability. However, many large academic medical centers care for patient populations similar to those seen at Barnes-Jewish Hospital. Third, some of the outcomes may be incompletely measured. While in-hospital vital status should always be available, some patients may not have a postoperative creatinine measurement available to distinguish whether AKI has occurred. However, AKI is expected to be extremely uncommon among patients without postoperative labs, minimizing the effect of this potential bias. Fourth, AKI is defined using creatinine only and not urine output, based on anticipated data availability. This may cause some cases of AKI to be missed. However, during ML model training, the incidence of AKI using this definition was compatible with the incidence of AKI reported in other studies. Finally, clinicians may give the ML display interface varying degrees of weight during case reviews randomized to ML assistance. Understanding this variability is of interest to the research team, which is why the case review form will ask the clinician how the ML display impacted their decision-making.

The expected result is that prediction accuracy for death and for AKI to be greater with ML assistance than without ML assistance. If the null hypothesis is rejected for only one of the two co-primary outcomes, this result will still be viewed as evidence that the ML assistance is beneficial. A possible unintended consequence would be if false negative ML predictions lead telemedicine clinicians to pay less attention to some patients who are actually at high risk for death or AKI. ^22^ Even if ACT clinicians monitor these patients less intensely, these patients will still receive standard-of-care monitoring and care by clinicians in the operating rooms. Thus, the reduction in telemedicine monitoring should not result in patient harm. The telemedicine clinicians supplement, but do not replace, standard care.

### Conclusion

Intraoperative telemedicine has the potential to improve postoperative outcomes if interventions can be targeted to patients most at-risk for complications, and ML may be able to help clinicians distinguish which patients those are. Periop ORACLE will test the hypothesis that anesthesiology clinicians in a telemedicine setting can predict postoperative complications more accurately with ML assistance than without ML assistance. By nesting this study within the ongoing TECTONICS randomized clinical trial of intraoperative telemedicine, the investigators will efficiently assemble a large dataset of clinician predictions that will be used to achieve the study objective. Successful completion of this study will help define the role of ML not only for intraoperative telemedicine, but for other risk assessment tasks before, during, and after surgery.

## Data availability

No data are associated with this article.

## Reporting guidelines

This protocol is presented according to the SPIRIT guidelines.

Open Science Framework: “Protocol for the Perioperative Outcome Risk Assessment with Computer Learning Enhancement (Periop ORACLE) Randomized Study”. https://doi.org/10.17605/OSF.IO/GC4ES. ^43^

Data are available under the terms of the Creative Commons Zero “No rights reserved” data waiver (CC0 1.0 Public domain dedication).
