# Supplementary material for: Protocol for the perioperative outcome risk assessment with computer learning enhancement (Periop ORACLE) randomized study
Source: F1000Res. 2022 Sep 29;11:653. Originally published 2022 Jun 14. [Version 2] doi: 10.12688/f1000research.122286.2 (PMC10397896; doi:10.12688/f1000research.122286.2)
Supplement: Peer review history

# Peer review: Reviewer response for version 2

**DOI:** 10.5256/f1000research.138973.r177469

**Published:** 2023-08-02

**Authors:** Pengbin Yin, Ming Chen, Yi Li

**Affiliations:** 1 Chinese PLA General Hospital, Beijing, China; 2 Department of Orthopedics, Chinese PLA General Hospital, Beijing, China

This protocol aimed to clarify the accuracy of machine-learning based prediction models over anesthesiology clinicians in assessing the risk of 30-day in-hospital mortality and AKI in adult surgical patients. This is a well-design protocol and of great clinical significance. However, in order to improve the manuscript's accessibility, readability, and overall quality, a few concerns are raised.

 1. Has this prediction model previously been tested for predictive performance in external validation?

 2. You mentioned the clinician selects their choice from a five-point ordered categorical scale (very low risk, low risk, average risk, high risk, and very high risk). What are the definitions of the five categories? Were all clinicians involved in the case review of this trial well-trained? Do these evaluation criteria remain consistent during all the practice process?

Is the study design appropriate for the research question?

Yes

Is the rationale for, and objectives of, the study clearly described?

Yes

Are sufficient details of the methods provided to allow replication by others?

Yes

Are the datasets clearly presented in a useable and accessible format?

Yes

Reviewer Expertise:

Clinical and basic research with focus on extracellular vesicles-related mechanisms discovery, targeted therapy development, biomarker identification, and prognosis improvement for ageing-related musculoskeletal conditions including fractures, osteoporosis, sarcopenia, etc.

We confirm that we have read this submission and believe that we have an appropriate level of expertise to confirm that it is of an acceptable scientific standard.

# Peer review: Reviewer response for version 2

**DOI:** 10.5256/f1000research.138973.r151957

**Published:** 2022-10-06

**Authors:** Michael Robert Mathis

**Affiliations:** 1 Department of Anesthesiology, University of Michigan Medical School, Ann Arbor, MI, USA

I have reviewed the revised manuscript and do not consider any additional revisions necessary. I approve the article for indexing.

Is the study design appropriate for the research question?

Yes

Is the rationale for, and objectives of, the study clearly described?

Yes

Are sufficient details of the methods provided to allow replication by others?

Yes

Are the datasets clearly presented in a useable and accessible format?

Yes

Reviewer Expertise:

NA

I confirm that I have read this submission and believe that I have an appropriate level of expertise to confirm that it is of an acceptable scientific standard.

# Peer review: Reviewer response for version 1

**DOI:** 10.5256/f1000research.134258.r143767

**Published:** 2022-07-22

**Authors:** Michael Robert Mathis

**Affiliations:** 1 Department of Anesthesiology, University of Michigan Medical School, Ann Arbor, MI, USA

This study is a protocol a sub-study nested within the TECTONICS RCT (assessing the impact of telemedicine approaches to preoperative risk assessment), seeking to understand the impact of machine-learning based prediction models to improve the accuracy of such preoperative risk assessments. 

 The study follows SPIRIT guidelines for reporting, and is overall well-written and of great clinical significance to anesthesiologists. Strengths of the study include its rigorous design; weaknesses include its single-center nature and perhaps limited exploration of unintended consequences of machine learning algorithm implementation. 

 Overall I believe this study will be an important scientific contribution to the field of anesthesiology, and look forward to its execution. However as the protocol is currently written, I have several critiques for the authors to address:

 1) There limitations of using solely a creatinine-based AKI endpoint (as opposed to creatinine + UOP + dialysis), but on balance I recognize the choice to focus on simply creatinine, given it is widely available within the EHR. However, there are other unusual circumstances that the authors might consider, that may lead to misguided conclusions if left unaddressed:

- a. How are patients with stage 4 or 5 CKD handled? Perhaps they are included, but it is misleading to think that AKI in such patients has anything to do with perioperative care, as most will develop AKI simply due to their native pathophysiology.
- b. If there is an EGFR-based cut-off for inclusion, how is EGFR computed? Would recommend the 2021 CKD-EPI formula which addresses racial bias.
- c. How are patients with no preoperative creatinine available handled?
- d. How are patients undergoing procedures which by definition impair renal function handled? (e.g. renal artery embolization; nephrectomy for RCC, etc.). 
- e. Are dialysis access procedures (e.g. AV fistula placements) included in this study?

2) I agree from both a study power / and clinical significance standpoint in using AKI Stage 1 or greater as AKI outcome (and of course mortality is more challenging to power); however you might consider breaking down AKI by stages as a secondary outcome (e.g. Stage 2 or greater; Stage 3 or greater), given their greater clinical significance (although even Stage 1 is important).

 3) It could be helpful to explore unintended consequences of the machine learning algorithm implementation. For example, workload/efficiency metrics may be useful to track: the authors may wish to track the total amount of ‘active review time’ used by clinicians in performing preoperative assessments with and without aid of the machine learning algorithm. If implemented in clinical care, there should be a plan (perhaps covered by the DSMB of the TECTONICS trial) to understand unexpected outcomes (for example, increased risk of stroke or MI, at the expense of decreased risk of AKI).

 4) As the AKI and mortality prediction algorithms are imperfect, do the clinicians performing preoperative risk assessments have insight into the performance characteristics of the algorithm (for example, do they know the algorithm’s positive predictive value, negative predictive value; perhaps Shapley values, AUROC, AUPRC, etc.), in order to better understanding exactly how much they should be trusting it? It may be interesting to study the impact of the machine-learning based risk assessments, for the cases in which the algorithm was *wrong*… in order to understand the potential impact of automation bias induced by machine learning algorithms on clinical decision-making.

 5) One of the major issues confronting ML-based algorithm support in healthcare decision-making is dataset shift – defined as an evolving mismatch between the data upon which the algorithm is trained upon, versus the data the algorithm is using in real-time to make predictions (PMID 34260843). Maintaining algorithm robustness over time remains very challenging, and decreases in algorithm performance can be seen whenever there are changes to practice patterns, EHR documentation patterns, or shifts in the patient population being studied. Although this study is limited by its single-center nature, it is not limited in its ability to assess temporal trends / algorithm de-tuning (and potential effects of algorithm re-tuning, if the authors wish to incorporate this into the study) over the time period studied. I would suggest assessing temporal trends in (i) AKI and mortality prediction algorithm performance, which if decreased may lead to a temporal trend in (ii) outcomes of this study. 

 6) Whereas the study protocol appropriately follows SPIRIT guidelines, when this study is executed and reported, this study will most likely need to follow DECIDE-AI guidelines (PMID 35585198), although I could be mistaken. The study team may wish to consider that downstream study now, and have the trial protocol written in such a way that the downstream study will be able to best adhere to DECIDE-AI reporting guidelines.

Is the study design appropriate for the research question?

Yes

Is the rationale for, and objectives of, the study clearly described?

Yes

Are sufficient details of the methods provided to allow replication by others?

Yes

Are the datasets clearly presented in a useable and accessible format?

Yes

Reviewer Expertise:

Machine learning applied to healthcare; anesthesiology; prediction modeling; large observational database research; acute kidney injury prediction.

I confirm that I have read this submission and believe that I have an appropriate level of expertise to confirm that it is of an acceptable scientific standard, however I have significant reservations, as outlined above.

# Response

**Published:** 2022-09-21

**Authors:** Bradley Fritz

**Affiliations:** Washington University in St. Louis, USA

Thank you for your thoughtful review of this protocol. We have revised the protocol to address your concerns, and we feel these changes have made the protocol stronger. Please see our point-by-point response below:

**COMMENT 1a. How are patients with stage 4 or 5 CKD handled? Perhaps they are included, but it is misleading to think that AKI in such patients has anything to do with perioperative care, as most will develop AKI simply due to their native pathophysiology.**

 Patients who are on dialysis preoperatively and patients with baseline creatinine level > 4.0 mg/dl will be excluded from the analyses of AKI, but other patients with CKD will be included. We agree that AKI will be highly prevalent among patients with CKD. Although some (many?) cases of AKI in this population will not be preventable, other cases will be driven at least partly by modifiable risk factors.

 All patients, including those on dialysis preoperatively and those with baseline creatinine > 4.0 mg/dl, will be included in the analyses of postoperative death. We have added a sentence to make this clearer:

*“Patients on dialysis preoperatively, undergoing dialysis access procedures, or with baseline creatinine > 4.0 mg/dl will be excluded from the AKI analysis but included in the death analysis.”* (Methods/Statistical Methods)

**COMMENT 1b. If there is an EGFR-based cut-off for inclusion, how is EGFR computed? Would recommend the 2021 CKD-EPI formula which addresses racial bias.**

 We agree that historically common EGFR equations can introduce racial bias, and we appreciate the reviewer’s suggestion for an alternative. As noted above, we will not use an EGFR-based cut-off for inclusion.

**COMMENT 1c. How are patients with no preoperative creatinine available handled?**

 If no preoperative creatinine was available, then the upper limit of the laboratory’s reference range (1.2 mg/dl) was used as the baseline. We felt this was a reasonable approximation because patients with abnormal renal function before surgery would most likely have a creatinine value available. We have added a sentence to make this clearer:

*“If no preoperative creatinine is available, then the upper limit of the laboratory’s reference range (1.2 mg/dl) will be used as the baseline.”* (Methods/Outcomes)

**COMMENT 1d. How are patients undergoing procedures which by definition impair renal function handled? (e.g. renal artery embolization; nephrectomy for RCC, etc.).**

 These patients will be included in the AKI analyses. Although it may frequently not be possible to prevent AKI in these situations, it may be possible to lessen its severity. Therefore, a clinician would want to be aware of the elevated risk in these patients.

**COMMENT 1e. Are dialysis access procedures (e.g. AV fistula placements) included in this study?**

 Patients undergoing dialysis access procedures will be excluded from the analyses of AKI. However, they will be included in the analyses of postoperative death. We have added a sentence to make this clearer:

*“Patients on dialysis preoperatively, undergoing dialysis access procedures, or with baseline creatinine > 4.0 mg/dl will be excluded from the AKI analysis but included in the death analysis.” *(Methods/Statistical Methods)

**COMMENT 2. I agree from both a study power / and clinical significance standpoint in using AKI Stage 1 or greater as AKI outcome (and of course mortality is more challenging to power); however you might consider breaking down AKI by stages as a secondary outcome (e.g. Stage 2 or greater; Stage 3 or greater), given their greater clinical significance (although even Stage 1 is important).**

 Thank you for this suggestion. We have added these as secondary outcomes:

*“Secondary outcomes will include AKI stage 2 or greater (creatinine increase to ≥ 2 times baseline within seven days) and AKI stage 3 (creatinine increase to ≥ 3 times baseline within seven days, an increase to ≥ 4.0 mg/dl, or initiation of renal replacement therapy).”* (Methods/Outcomes)

**COMMENT 3. It could be helpful to explore unintended consequences of the machine learning algorithm implementation. For example, workload/efficiency metrics may be useful to track: the authors may wish to track the total amount of ‘active review time’ used by clinicians in performing preoperative assessments with and without aid of the machine learning algorithm. If implemented in clinical care, there should be a plan (perhaps covered by the DSMB of the TECTONICS trial) to understand unexpected outcomes (for example, increased risk of stroke or MI, at the expense of decreased risk of AKI).**

 We agree it would be insightful to investigate the time spent reviewing each case. If the machine learning algorithms have an impact on active review time, then the impact could plausibly be in either direction: clinicians may spend less time in the patient’s chart for ML-assisted cases because the algorithms have expedited the risk assessment process, or they may spend more time because the algorithms have pointed out concerns the clinicians otherwise would not have noticed. We have added this to the data collection and analysis plans:

*“The amount of time the clinician in the ACT spends completing each case review will be retrieved from the Epic audit log.”* (Methods/Data collection)

*“To estimate the effect of the ML predictions on case review efficiency, chart review duration (approximated using the time the Epic chart was open, retrieved from the audit log) will be compared between case reviews performed with ML assistance and those performed without ML assistance, using either an unpaired T test or Wilcoxon rank sum test as appropriate.”* (Methods/Statistical methods)

 Information from the case reviews performed in ORACLE will only be communicated to bedside clinicians in the operating room if the patient is in the intervention arm of the parent TECTONICS trial. (All patients in ORACLE are also enrolled in TECTONICS.) The TECTONICS trial has a data safety monitoring committee that reviews adverse events.

COMMENT 4. As the AKI and mortality prediction algorithms are imperfect, do the clinicians performing preoperative risk assessments have insight into the performance characteristics of the algorithm (for example, do they know the algorithm’s positive predictive value, negative predictive value; perhaps Shapley values, AUROC, AUPRC, etc.), in order to better understanding exactly how much they should be trusting it? It may be interesting to study the impact of the machine-learning based risk assessments, for the cases in which the algorithm was *wrong*… in order to understand the potential impact of automation bias induced by machine learning algorithms on clinical decision-making.

 We have added a new paragraph to explain what pieces of meta-data are provided along with the predictions:

*“For each patient, the machine learning predictions are presented in the form of predicted probabilities of each outcome (e.g., 4.5% chance of AKI). In addition, a list of features contributing most to the prediction is shown, along with Shapley values. During the focus group meetings, most users said they would find it overwhelming to see confidence intervals around the predicted probabilities. A fact sheet about each model is available on demand, including the receiver operating characteristic curve, precision-recall curve, and calibration curve.”* (Methods/Interventions – Machine Learning Algorithms)

 To address the impact of “wrong” predictions, we have added a sensitivity subgroup analysis:

*“To examine the potential impact of inaccurate ML predictions, we will conduct the following sensitivity analysis. First, the predicted probabilities output by the ML algorithms will be converted to dichotomous predictions by using the cutoff value that maximizes the Youden index. Second, the cases will be categorized as having correct or incorrect ML dichotomized predictions. Finally, the primary analysis will be repeated in the subgroup with correct ML predictions and in the subgroup with incorrect ML predictions.”* (Methods/Statistical methods)

**COMMENT 5. One of the major issues confronting ML-based algorithm support in healthcare decision-making is dataset shift – defined as an evolving mismatch between the data upon which the algorithm is trained upon, versus the data the algorithm is using in real-time to make predictions (PMID 34260843). Maintaining algorithm robustness over time remains very challenging, and decreases in algorithm performance can be seen whenever there are changes to practice patterns, EHR documentation patterns, or shifts in the patient population being studied. Although this study is limited by its single-center nature, it is not limited in its ability to assess temporal trends / algorithm de-tuning (and potential effects of algorithm re-tuning, if the authors wish to incorporate this into the study) over the time period studied. I would suggest assessing temporal trends in (i) AKI and mortality prediction algorithm performance, which if decreased may lead to a temporal trend in (ii) outcomes of this study.**

 This is an important point that we had considered but failed to mention in the previous version of the protocol. At the time the study was initiated, we were utilizing models that had been trained on a retrospective cohort of patients who had surgery at Barnes-Jewish Hospital between 2012 and 2016. Approximately six months after study initiation, we retrained the models using patients who had surgery between 2018 and 2020. We have added two new paragraphs to the methods section to explain this clearly:

*“The machine learning models used in this study were originally trained and validated on a retrospective cohort of approximately 110,000 adult patients who underwent surgery with general anesthesia at Barnes-Jewish Hospital between 2012 and 2016. Input features included demographic characteristics, comorbid conditions, preoperative vital signs, surgical service, functional capacity as documented during the preoperative assessment, and most recent values of selected laboratory tests. A random forest model was implemented in scikit-learn. In the holdout validation cohort of 21,171 patients, the incidence of postoperative death was 2.2% and the model predicted this outcome with receiver operating characteristic area under curve (AUC) of 0.939 and precision-recall AUC of 0.161. The incidence of postoperative AKI was 6.1% and the model predicted this outcome with receiver operating characteristic AUC of 0.799 and precision-recall AUC of 0.275.*

*In February 2022, the models were retrained using a newer cohort of 84,455 patients who underwent surgery with general anesthesia at Barnes-Jewish Hospital between 2018 and 2020. This time period was after the hospital had transitioned from its previous electronic health record systems (including MetaVision as its anesthesia information management system) to Epic (Epic, Verona, WI). Input features were the same as the previous models, with the addition of the planned surgical procedure text field. A regularized logistic regression was used to predict the outcome from the words in the planned surgical procedure text field. This was used to initialize a gradient boosted decision tree, which was trained using the remaining features. Hyperparameters were selected by 10-fold cross validation. Models were implemented in XGBoost. In the holdout validation cohort of 16,891 patients, the incidence of postoperative death was 1.9% and the model predicted this outcome with receiver operating characteristic AUC of 0.91 and precision-recall AUC of 0.27. The incidence of postoperative AKI was 13.3% and the model predicted this outcome with receiver operating characteristic AUC of 0.90 and precision-recall AUC of 0.66.”* (Methods/Interventions – Machine Learning Algorithms)

 To examine the effects of dataset shift on model performance and trends over time, we will report the prospective performance (i.e., AUC) of each model for each month of the study. To examine the effects of model retraining, we will also conduct subgroup analyses repeating the primary analyses in the subgroups who had surgery before and after the retraining event. We have added these steps to the statistical methods:

*“To examine the effects of dataset shift and model retraining, the prospective performance of the models will be reported for each month of the study, and sensitivity analyses will be conducted in the subgroups who had surgery before and after the February 2022 retraining event.”* (Methods/Statistical methods)

**COMMENT 6. Whereas the study protocol appropriately follows SPIRIT guidelines, when this study is executed and reported, this study will most likely need to follow DECIDE-AI guidelines (PMID 35585198), although I could be mistaken. The study team may wish to consider that downstream study now, and have the trial protocol written in such a way that the downstream study will be able to best adhere to DECIDE-AI reporting guidelines.**

 Thank you for this suggestion—we agree the study results will be reported according to DECIDE-AI guidelines. To make it easier for the results manuscript to adhere to DECIDE-AI guidelines, we have expanded the methods section of this protocol to add details that are required by the DECIDE-AI guidelines. In particular, we have broken up the Intervention subsection into two new sections describing the AI system (checklist item 4) and describing the implementation (checklist item 5), which are mentioned in our responses to your earlier comments. In addition, we have added statistical methods that will allow us to report on human-computer agreement (checklist item 12):

*“To examine human-computer agreement, the proportion of cases for which the clinician reported being surprised by the ML prediction will be determined. Among those cases for which the clinician was surprised, the proportion for which the clinician self-reported agreeing or disagreeing with the ML prediction will be determined.” *(Methods/Statistical methods)
